# The complete chloroplast genome sequence and phylogenetic analysis of *Nanhaia speciosa* (Fabaceae)

**DOI:** 10.1080/23802359.2021.2008828

**Published:** 2022-01-27

**Authors:** Yufei Xiao, Mimi Li, Lianxiang Zhong, Yufeng Qin, Xiaoning Zhang, Qiulan Wei, Zihai Qin, Ye Zhang, Bowen Chen

**Affiliations:** a Guangxi Forestry Research Institute, Nanning, China; b Institute of Botany, Jiangsu Province and Chinese Academy of Sciences, Nanjing, China; c The Jiangsu Provincial Platform for Conservation and Utilization of Agricultural Germplasm, Nanjing, China

**Keywords:** Chloroplast genome, Fabaceae, IR loss, Leguminosae, *Nanhaia speciosa*

## Abstract

*Nanhaia speciosa* (Fabaceae) is a woody perennial vine used as an important traditionally Chinese medicine. In this study, the complete chloroplast genome of *Nanhaia speciosa* was sequenced and assembled. The chloroplast genome of *N. speciosa* was 132,551bp in length including only one copy of the inverted repeat (IR). It encoded a total of 110 genes, containing 76 protein-coding genes, 30 tRNA and 4 rRNA. The overall GC content was 34.1%. Phylogenetic analysis using a matrix of 69 protein-coding genes illustrated that *N. speciosa* is most closely related to *Wisteriopsis reticulata* of tribe Wisterieae.

*Nanhaia speciosa* (Champ. ex Benth.) J.Compton & Schrire (2019), commonly name Niudali, is a woody perennial vine belonging to pea family (Fabaceae) distributed mainly in south China and Vietnam (Wei and Pedley 2010). The tuberous roots are used as a traditionally Chinese medicine with hepatoprotection, cough suppressants & expectorant and anti-asthmatic, increasing immunity effects (Yao et al. 2016; 2021). The wild resource of *N. speciosa* have been sharply reduced in recent year by overexploitation. Furthermore, the taxonomy of *N. speciosa* has been controversial. It was previously treated as *Millettia speciosa* Champ. ex Benth and placed in the *Callerya* group of tribe Millettieae (Wei et al. 2010). Now it transferred to the new genus *Nanhaia* of tribe Wisterieae because of differ in its densely pubescent ovaries, larger flower, persistent of floral bracts and gibbosities (Compton et al. 2019). The morphological characters indicated that it is closely related to the genus *Wisteriopsis* (Compton et al. 2019). Therefore, the chloroplast genomic information of *Nanhaia speciosa* presented here is valuable for future classification, phylogenetic and evolution studies in the family Fabaceae.

The fresh mature and healthy leaves of *Nanhaia speciosa* was collected from Guangxi Forestry Research Institute (22°55'30"N, 108°21′ E) and the voucher specimen was deposited at Guangxi Forestry Research Institute with number 20210317004 (contact: Bowen Chen, e-mail: bwchen_gfri@163.com). Total genomic DNA was extracted by modified CTAB (hexadecyltrimethylammonium bromide) method (Doyle and Doyle 1987). A paired-end sequencing library was generated using NEB Next® Ultra DNA Library Prep Kit for Illumina (NEB, USA) following manufacturer’s instructions with an insert size of 300 bp. We sequenced the library on an Illumina Hiseq X-ten platform (San Diego, USA) at Novogene Biotech Co., Ltd. (Beijing, China) and at least 2.49 GB of raw sequencing data were obtained.

High-quality reads were assembled into chloroplast genome using the software NOVOPlasty 4.3.1 (Dierckxsens et al. 2017) with *Callerya nitida* (MT120748) as a reference. The genome annotation was applied in GeSeq (Tillich et al. 2017) and manually adjusted the start/stop codons and/or intron/exon boundaries in Geneious 11.1.5 ( Kearse et al., 2012). The final complete chloroplast genome sequence was submitted to GenBank databases (National Center for Biotechnology Information, NCBI) through Bankit (https://submit.ncbi.nlm.nih.gov/about/bankit/) with accession number MZ028462.

The chloroplast genome of *N. speciosa* was 132,551bp in length including only one copy of the inverted repeat (IR). It encoded a total of 110 genes, containing 76 protein-coding genes, 30 tRNA and 4 rRNA. The overall G + C content was 34.1%. Only one gene (*ycf*3) had two introns, while six tRNA(*trn*I-GAU, *trn*K-UUU, *trn*A-UGC, *trn*G-UCC, *trn*V-UAC, *trn*L-UAA) and 10 protein-coding genes (*rps*12, *rpo*C1, *ndh*A, *ndh*B, *rpl*2, *rpl*16, *pet*B, *atp*F, *clp*P, *pet*D) had one intron. Three genes (*rps*16, *rpl*22 and *inf*A) were lost in *N. speciosa*, which was the same as that reported previously for the other legume species (Oyebanji et al. 2020).

A total of 16 cp genome sequences were used for the phylogenetic analysis with 15 plastome data downloaded from GenBank. We performed the alignment in software MAFFT 7.409 (Katoh and Standley 2013) using 69 protein-coding genes. *Glycyrrhiza glabra* (NC_024038) and *G. uralensis* (KU862308) were defined as outgroups. The phylogenetic tree was constructed by the GTR + GAMMA nucleotide substitution model implemented in RAxML with 1000 bootstrap replicates (Stamatakis 2014). Based on the phylogenetic trees, most of nodes were well resolved with strongly bootstrap values of 72 ∼ 100% that provided robust topological structure of the selected taxa in Fabaceae. The results illustrated that *N. speciosa* is sister to *Wisteriopsis reticulata* (MT120816) of tribe Wisterieae (Figure 1) with a 100% bootstrap value, which is consistent with previous studies using morphological traits and molecular fragments (Compton et al. 2019). The present study suggested that the chloroplast genome sequences will provide a theoretical basis for future studies of Fabaceae in the evolution and phylogenetic relationships.

**Figure 1.**
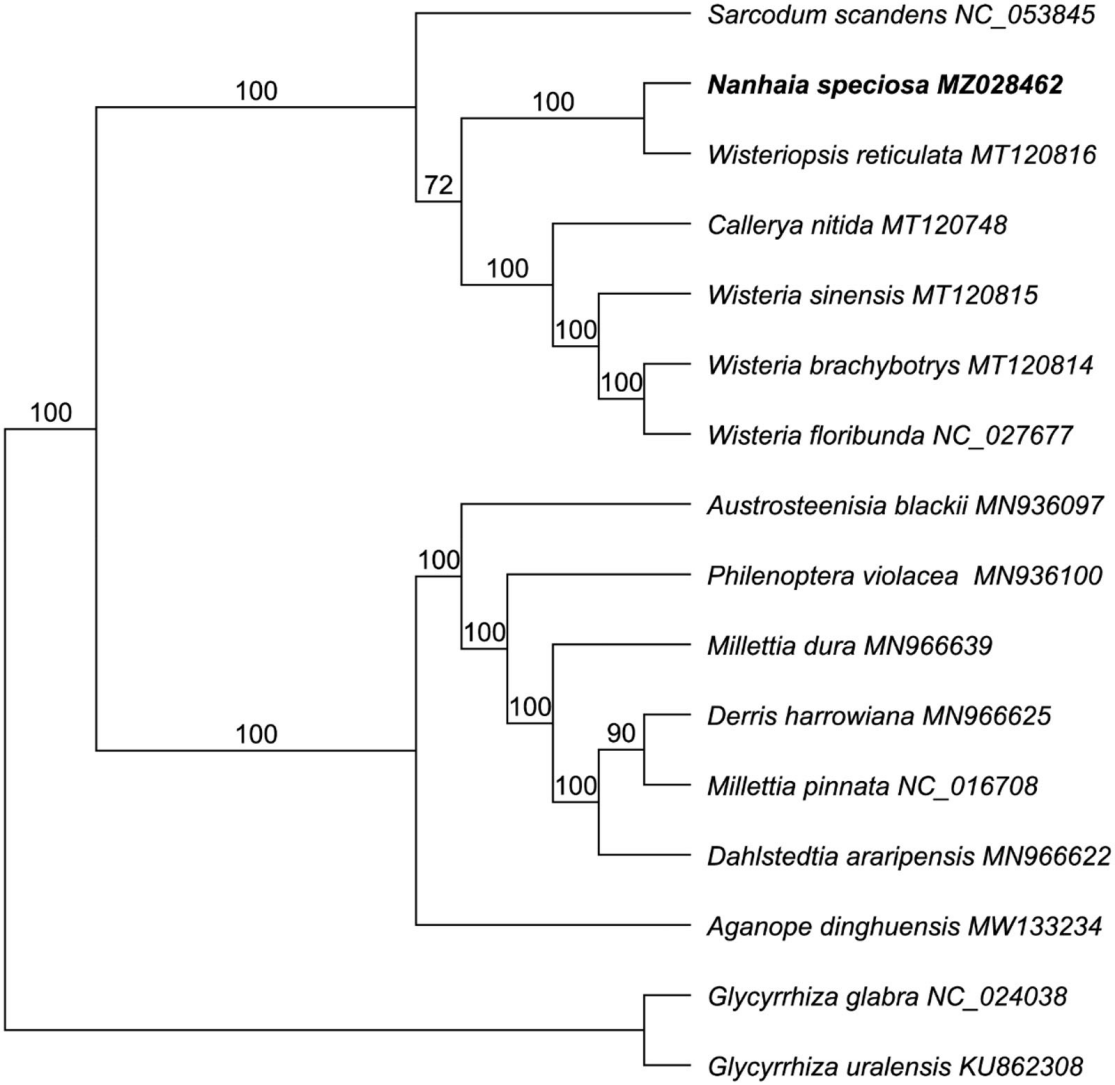
Maximum-likelihood (ML) tree *N. speciosa* and other 15 species. Numbers on the branch indicated the bootstrap values after 1000 replicates.

## Data Availability

The genome sequence data that support the findings of this study are openly available in GenBank of NCBI at (https://www.ncbi.nlm.nih.gov/) under the accession no. MZ028462. The associated BioProject, SRA, and Bio-Sample numbers are PRJNA743413, SRR15032818 and SAMN20034749, respectively.
